# Evaluation of Tissue Expression of Vaspin and Serum Vaspin Concentration as a Prognostic and Risk Factor in Endometrial Cancer

**DOI:** 10.3390/cells11203196

**Published:** 2022-10-11

**Authors:** Mateusz Kozłowski, Dominika Pietrzyk, Małgorzata Rychlicka, Katarzyna Piotrowska, Katarzyna Nowak, Sebastian Kwiatkowski, Aneta Cymbaluk-Płoska

**Affiliations:** 1 Department of Gynecological Surgery and Gynecological Oncology of Adults and Adolescents, Pomeranian Medical University in Szczecin, al. Powstańców Wielkopolskich 72, 70-111 Szczecin, Poland; 2 Department of Nursing, Pomeranian Medical University, 71-210 Szczecin, Poland; 3 Department of Physiology, Pomeranian Medical University in Szczecin, al. Powstańców Wielkopolskich 72, 70-111 Szczecin, Poland; 4 Department of Obstetrics and Gynecology, Pomeranian Medical University in Szczecin, al. Powstańców Wielkopolskich 72, 70-111 Szczecin, Poland

**Keywords:** vaspin, endometrial cancer, prognostic factor, risk factor

## Abstract

Adipose tissue is a multifunctional endocrine organ. One of the biologically active substances is vaspin, which is part of the serpin family. The purpose of the following study is to determine the possibility of using vaspin as a prognostic and risk factor in endometrial cancer. The study included 127 patients with abnormal uterine bleeding. To determine the value of adipokine, the study used Kaplan-Meier curves to estimate patients survival. Univariate and multivariate analyses were performed simultaneously using the Cox regression model. Tissue expression of vaspin was assessed in patients from the study group (endometrial cancer) and the control group (non-cancerous). We found that higher levels of vaspin are found in obese people, with lower staging (FIGO I and II), lower grading (G1), no LVSI metastases and no lymph node metastases. Higher serum vaspin levels are an independent protective factor for endometrial cancer. We concluded that endometrial cancer patients with serum vaspin concentrations above the median have longer DFS compared to patients with concentrations below the median. Considering multivariate analysis, vaspin concentrations above the median are independent favourable prognostic factors for endometrial cancer. Tissue expression of vaspin cannot be a histological marker to distinguish between cancer and non-cancerous lesions and between different grading levels.

## 1. Introduction

Endometrial cancer (EC) is one of the most common gynaecological cancer in the developed world [1]. It is the sixth most common cancer in women, according to the World Cancer Research Fund [2]. Furthermore, in terms of mortality, it is the seventh cancer in Poland (3.6% of deaths) [3]. The majority of endometrial cancer diagnoses are considered to be hormonally driven, emphasizing a key role for estrogen signaling in the disease, unbalanced by progesterone [4]. In particular, type 1 of endometrial cancer typically develops on the basis of atypical endometrial hyperplasia. On the contrary, type 2, which belongs to minorities; it occurs mainly in older postmenopausal women, characterized by a worse prognosis and is ER- (no expression for estrogen receptor). The most commonly described molecular alterations are mutations of PTEN (phosphatase and tensin homolog), KRAS (Kirsten rat sarcoma virus), *TP53* (tumor protein P53) and overexpression of L1CAM (L1 cell adhesion molecule), expression of ER and PR (estrogen and progesterone receptors) [5]. Around postmenopausal age, infertility or estrogen-secreting ovarian tumors are relevant risk factors for EC [6]. In addition, there is an undeniable correlation between the increasing incidence of metabolic diseases and the rising frequency of endometrial cancer diagnoses [7]. In recent years, increasing attention has been paid to obesity, metabolic syndrome and type 2 diabetes [8]. Adipose tissue is known as an endocrine organ because of its association with adipokines secretion such as vaspin, which belongs to the serine protease inhibitor family [9]. Obese women have a 3-fold increased risk of developing endometrial cancer [4]. It can be noted the link between significantly higher amount of vaspin in obese patients and pathologies connected with hormonal imbalance [10]. Regarding the pathogenesis, the increased expression of the receptor for insulin and IGF-1 (insulin-like growth factor) contributes to anabolic effects on the endometrium and leads to its hypertrophy [11]. High hormonal and enzymatic activity in visceral fat, translates into a state of hyperinsulinemia [12] and the resulting hyperandrogenism and hyperestrogenism [13,14]. Androstendione is the main male sex hormone in the blood of obese women subject to aromatization to estrogen in overdeveloped adipose tissue [15,16]. In the course of PCOS (polycystic ovary syndrome) can be a state of hyperprolactinemia, which stimulates adrenal androgen production. Furthermore, reduced SHBG (sex hormone binding globulin) levels in obese women increase the bioavailability of androgens, leading to the progression of ovarian pathology and increased levels of biologically active estrogens [17]. In the course of primary hypothyroidism, a state of hyperprolactinemia also develops. As a result of decreased production of thyroxine (T4) and triiodothyronine (T3) through a negative feedback mechanism, TRH (thyrotropin-releasing hormone) and TSH (thyroid-stimulating hormone) secretion are increased. The role of estrogen as a mitogenic, but also mutagenic factor is also known. Genotoxic metabolites react with DNA leading to the accumulation of breaks and genetic instability [18]. Much evidence points to vaspin’s involvement in the increase in insulin sensitivity of adipose tissue and normalizing plasma glucose levels [19,20,21]. Vaspin attenuates phosphorylation of the insulin receptor and its product, resulting in inhibition of insulin receptor (IRS2) activity. Moreover, studies have shown vascular smooth muscle proliferation and chemokinesis can be reduced by blocking insulin receptor signaling and the NF-κB (nuclear factor kappa-light-chain-enhancer of activated B cells) signaling pathway [22].

In the following study, we present the correlation of vaspin concentrations with various risk factors. The purpose of this study is to present the possibility of using vaspin as a prognostic and risk factor among patients with endometrial cancer.

## 2. Materials and Methods

### 2.1. Participation in the Study

The study included 127 patients of the Department of Gynecological Surgery and Gynecological Oncology of Adults and Adolescents Pomeranian Medical University in Szczecin, Poland. All the patients signed informed consent to participate in the study, which was approved by the Ethical Committee of the Pomeranian Medical University. Inclusion criteria for the study were abnormal uterine bleeding and/or abnormal ultrasound images, and patient consent. The exclusion criteria were: lack of patient consent, patients with histopathologically diagnosed endometrial hyperplasia, acute inflammation, other malignancies, collagenosis, chronic kidney disease, cirrhosis, treatment with biological agents and immunotherapy.

### 2.2. Division of Patients into Study and Control Groups

A division was made into two main groups according to histopathological diagnosis after endometrial biopsy, uterine curettage or hysteroscopy. In the case of surgical treatment, it was adjusted according to the clinical stage and histopathological differentiation of the tumor lesions.

Group A—Patients with benign changes, *n* = 62, subgroups:

- A1 *n* = 30: endometrial polyps
- A2 *n* = 32: uterine myomas

Group B—Patients with endometrial cancer, *n* = 65, subgroups (histopathological type):

- B1 Type I cancer (endometrial endometrioid adenocarcinoma)
- B2 Type II cancer (serous endometrial carcinoma, squamous adenocarcinoma and clear cell carcinoma)

Patients were divided into three groups depending on BMI (body mass index):

The following equation was used to determine BMI

BMI = Weight [kg]/Height^2^ [m]

1. BMI 20–25 kg/m^2^, *n* = 14 patients
2. BMI 25–30 kg/m^2^, *n* = 25 patients
3. BMI >30 kg/m^2^, *n* = 26 patients

Patients were divided into two groups depending on WC index (waist circumference):

1. WC > 100 cm, *n* = 42
2. WC < 100 cm, *n* = 23

Patients were divided into two groups according to the WHR index (waist-hip ratio):

1. WHR > 0.8, *n* = 38
2. WHR < 0.8, *n* = 27

Patients were divided into groups according to the presence or absence of the following risk factors for endometrial cancer:

Due to the presence of arterial hypertension (HA > 140/90 mmHg):

1. HA—no, *n* = 24
2. HA—yes, *n* = 41

Due to the presence of type 2 diabetes (DM type 2):

1. DM type 2—yes, *n* = 40
2. DM type 2—no, *n* = 25

Due to the fasting blood glucose:

1. incorrect >110 mg%, *n* = 48
2. correct <110 mg%, *n* = 17

Due to the hormonal status:

1. premenopausal state, *n* = 13
2. postmenopausal state, *n* = 52

Due to the thyroid diseases:

1. presence, *n* = 38
2. absence, *n* = 27

Due to use of hormone replacement therapy:

1. yes, *n* = 37
2. no, *n* = 28

### 2.3. Pre-Laboratory Sample Preparation

Serum vaspin levels were determined in all patients, and postoperative material in the form of paraffin blocks was analyzed. Slide preparations were also prepared. In the study group, after surgical treatment, the analysis was performed taking into account the histopathological differentiation of the tumor (grading) and the clinical stage of the tumor (staging). Patients with endometrial cancer, were divided into three stages of tumor grading: G1 = 18, G2 = 32, G3 = 15. The clinical stage according to FIGO (International Federation of Gynecology and Obstetrics) was also determined (staging):

1. FIGO I and II, *n* = 46 patients
2. FIGO III and IV, *n* = 19 patients

Division of endometrial cancer patients is presented in Table 1.

### 2.4. The Identification of Vaspin by Immunoenzymatic ELISA

Biochemical determinations were performed after centrifugation of the collected blood and freezing of the resulting serum in Eppendorf-type containers stored at −80 °C. Vaspin was quantified in serum by immunoenzymatic ELISA-multiplex fluorescence (Luminex Corporation, Austin, TX, USA) using a commercial Bio Plex Pro RBM Human Metabloic Panel 2 (Biorad, Hercules, CA, USA). Fifty microliters of antibody capturing capsule solution was added to each well of the test plate, and the plate was washed twice with 100 microliters of wash buffer. Fifty microliters of buffer was added to each blank, standard and test samples, and the plate was incubated for one hour by programming automatic mixing of the substances. The substances were then mixed three times using a hand magnet. A cocktail of detection antibodies was added by pipette to each well, and the plate was sealed and incubated at room temperature for 30 min. After washing with 50 microliters of wash mixture, streptavidin and phycoerythrin were added and the plate was incubated in the dark for 10 min. The plate was then read and analyzed on a Luminex analyzer. Vaspin concentration was read from the curves by comparing to standard curves and extracting the median fluorescence intensity as a function of vaspin concentration.

### 2.5. Immunohistochemical Analysis

Immunohistochemical analysis of vaspin was carried out by rehydrating deparaffinized uterine scrapings (3 μm thick) and performing thermal epitope recovery in a microwave oven in solution recovery buffer pH = 6 (DAKO Agilent, Santa Clara, CA, USA). After cooling to room temperature (RT), the slides were incubated with 0.3% oxidized water solution, then washed twice with PBS (phosphate-buffered saline) and further incubated with 2.5% horse serum (Vector Laboratories Newark, CA, USA). After incubation with serum, the slides were incubated with rabbit primary antibodies against human vaspin for one hour at RT. After washing in PBS (EURx, Gdańsk, Poland), immune reactions were visualized using ImmPRESS UNIVERSAL REAGENT and Vector Nova RED Substrate KIT FOR PEROXIDASE (Vector Laboratories Newark, CA, USA) according to the protocol posted by the manufacturer. The primary PBS antibody was substituted as a negative control in the sample. Positive staining was determined by visual identification of yellow-brown pigmentation by light microscopy. Images were collected using an Olympus IX81 inverted microscope (Olympus, Germany) with a color camera and CellSens image processing software 1.16 (Olympus, Germany).

### 2.6. Statistical Analysis

Statistica version 10 PL software was used for statistical analyses. The following characteristics were used to provide descriptive characteristics describing the patient group: minimum, maximum, data range and median values. A comparison of the two structure indicators (i.e., percentages) was also made. Using the Shapiro-Wilk test, we examined whether the variables under study had a normal distribution. The variables do not have a normal distribution, the exception being the variable age for the entire given population, whose distribution is a normal distribution (W = 0.97863, *p* = 0.60221). Therefore, the analysis used non-parametric methods to test the relationship (Spearman’s rank correlation coefficient) and non-parametric significance tests. The nonparametric Man-Whitney U test of significance for independent samples was used to verify the hypothesis that the distributions of the two variables come from the same populations. A non-parametric Kruskal-Wallis significance test (e.g., by clinical stage or histopathologic differentiation) was used to verify the hypothesis that the distributions of more than two variables are from the same populations. POST-HOC tests were used to examine which populations differed. Kaplan-Meier curves were used to perform imaging survival analysis, and the log-rank test was used to characterize the effect on disease-free time (DFS) and overall survival (OS) of the new biomarker vaspin under investigation. Two analyses were also performed: univariate and multivariate analysis using the Cox regression model. The parameters included in the multivariate Cox analysis included age, staging, grading, and the median, 75th percentile, and 95th percentile of a new marker, vaspin (these were preoperative values). A value of *p* < 0.05 was considered an indicator of statistical significance.

## 3. Results

### 3.1. Characteristics of the Study Group

The largest percentage of patients qualified for the study were patients who were divorced (34%). In contrast, the smallest percentage were single patients (17%). Statistically significant differences were not revealed between patients according to place of residence and education. The largest number of patients resided from cities with less than 100,000 inhabitants (45%). Hypertension and type 2 diabetes did not differ between the study groups. Among the patients recruited for the study, more were postmenopausal. Results are presented in Table 2.

In the group of patients with endometrial cancer, 42.9% were single; this percentage looked like 47.6% and 9.5% for endometrial polyps and uterine myomas respectively. Statistically significant correlations were only noted between patients with uterine myomas in the percentage between single and married women (*p* = 0.032) and in patients with endometrial polyps between the same studied characteristics (*p* = 0.045). The highest percentage of divorced and widowed patients occured in the diagnosis of endometrial cancer endometrium and were, respectively: 48.8% and 50%. The percentage of endometrial cancer patients is 46.9% for the group of patients with vocational education, while the percentage of endometrial cancer patients with secondary and higher education were as follows 51.7%, 54.1%. Only in the study group of patients with endometrial cancer were no statistically significant differences found, while they were noted taking into account the level of education among patients with endometrial polyps and uterine myomas. The percentage of patients with endometrial cancer was similar in patients living in countryside and cities with less than 100,000 inhabitants (51.2%, 43.9%). Statistically significant differences were not revealed in the group of patients with endometrial cancer from cities with less or more than 100,000 inhabitants. In the study group of patients with endometrial cancer, there were differences in the percentage of patients with diagnosed hypertension compared to patients without hypertension (*p* = 0.033). In the control group, patients with endometrial polyps also showed differences in the percentage of patients without hypertension to patients with hypertension and were: 30.5% to 17.6%. Differences in the incidence of type 2 diabetes in the study and control groups was not statistically significant. In the study group with endometrial cancer, it was 51.4% for patients diagnosed with diabetes mellitus type 2, while it was 51% for patients without type 2 diabetes. In the group of patients with endometrial cancer and endometrial polyps, statistically significant differences were found between the percentages of premenopausal versus postmenopausal patients. In the study group, the percentage ratio were: 32.5% and 59.8%. Statistically significant correlations were also noted in the group of patients with endometrial polyps, for premenopausal patients 32.5% versus postmenopausal patients 19.5% (*p* = 0.048).

In all the patient groups considered statistically significant differences were revealed between the number of patients with incidence of thyroid disease. However, patients with endometrial cancers exhibited significantly greater percentage of patients without concomitant diseases of the thyroids gland and were as follows: 64.2% to 46.9% (*p* = 0.042). In contrast, among patients in the control group there was the higher percentage of patients with thyroid disease: 27.2% for patients with endometrial polyps and 25.9% with uterine myomas vs. patients without concomitant thyroid disease: 19% and 16.8%. The percentage of patients in the control group with endometrial polyps was similar within each range of BMI. In contrast, In the study group of endometrial cancer patients, the lowest percentage of patients had a was with a normal weight, relatively close to overweight patients (*p* = 0.031). However, patients from the endometrium cancer group having WC below 100 compared to patients with WC above 100 exhibited statistically significant correlations (*p* = 0.006). In the control group with endometrial polyps and uterine myomas, the percentages were higher for patients with WC below 100 and were respectively: 35.8% and 29.9%. Results are presented in Table 3.

### 3.2. Evaluation of Serum Vaspin Levels in Relation to Risk Factors for Endometrial Cancer

**Figure 1:**
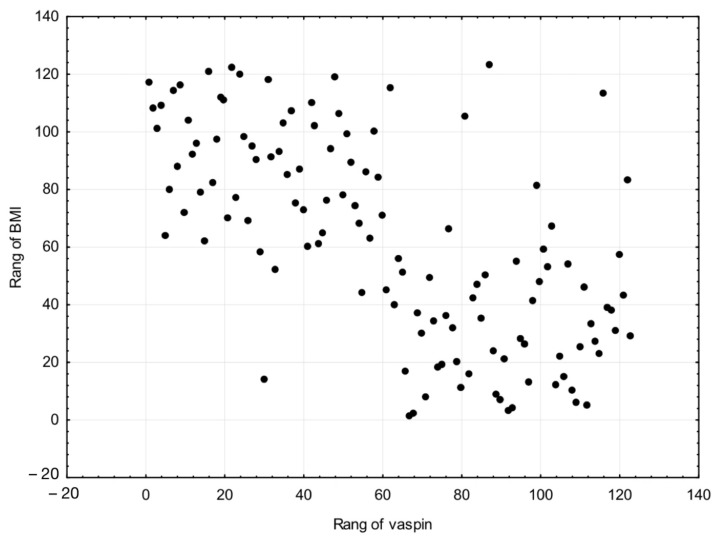
A strong correlation was found between BMI and serum vaspin levels (r = 0.658).

The median serum concentration of vaspin in normal weight patients was 2.8 ng/mL, which was statistically significantly lower than that of overweight patients at 3.5 ng/mL. The level of statistical significance was *p* = 0.038. There were significant differences in concentrations between overweight and obese patients (*p* = 0.012). As for overweight patients, the serum was 3.5 ng/mL, which was lower than the median serum vaspin concentrations of obese patients of 5.2 ng/mL. With regard to the median serum concentration of vaspin in normal weight patients we noted the level 2.8 ng/mL, which was significantly lower compared to median 5.2 ng/mL of obese patients. The difference between the serum concentrations was *p* = 0.004. Since the distribution of BMI is highly asymmetric, we used to calculate the relationship between BMI and vaspin by Spearman’s correlation. A strong correlation was found (r = 0.658). Results are presented in Table 4 and Figure 1.

Statistically significant correlations were also noted between patients with a waist circumference below and above 100 cm (*p* = 0.022). The median value of serum vaspin concentrations was higher among patients with waist circumference above 100 cm (5.5 ng/mL) compared to the patients with waist circumference below 100 cm (3.5 ng/mL). With regard to the median serum concentration of vaspin was lower among patients with hypertension (3.7 ng/mL) compared to the group of patients with proper blood pressure (4.1 ng/mL). This result was not statistically significant. Statistically significant differences were not revealed between median concentration of serum vaspin in the group of patients diagnosed with type 2 diabetes (5.0 ng/mL) versus patients without this chronic disease (5.1 ng/mL). Results are presented in Table 5.

### 3.3. Evaluation of Serum Vaspin Levels in Relation to Endometrial Cancer Staging and Grading

The median serum concentration of vaspin in patients with FIGO I, II was 1.5 ng/mL along with a range of values from 0.4 to 3.8 ng/mL, while in patients with FIGO III, IV was 1.0 ng/mL (range of values from 0.1 to 2.6 ng/mL). Statistically significant correlations were noted between patients with low and high tumor stage at *p* = 0.041. Median concentration of serum vaspin in G2 patients was 1.6 ng/mL along with a range of values from 0.8 to 3.3 ng/mL compared to lower median levels of vaspin in G3 patients (1.1 ng/mL) along with a range of values from 0.1 to 2.5 ng/mL. Result was not statistically significant for patients with medium and low histopathological differentiation of the tumor. Significantly higher values of serum vaspin were demonstrated in cases of patients with G1 endometrial cancer compared to serum vaspin levels in patients with G3 endometrial cancer (*p* = 0.049).

Considering to the depth of myometrial infiltration, we found the median value of serum vaspin levels in patients with superficial infiltration of the uterine myometrium at the level of 2.2 ng/mL along with a range of values from 0.4 to 4.1 ng/mL, while for deep infiltration of the myometrium, the average concentration is 2.0 ng/mL (range of values from 0.3 to 3.9 ng/mL). Statistically significant differences were not revealed between patients with different depths of infiltration of the myometrium. With regard to vaspin, we noted significantly lower serum concentrations in the presence of lymphovascular space invasion (LVSI metastases) in patients with cancer of the endometrium was 1.8 ng/mL along with a range of values from 0.3 to 4.1 ng/mL, while in patients without LVSI metastases was 2.8 ng/mL (range of values from 0.5 to 4.4 ng/mL). This result was statistically significant *p* = 0.037. However, statistically significant differences were not revealed between median concentration of serum vaspin in the group of patients with the current angioinvasion (1.5 ng/mL), along with a range of values of 0.1–3.5 ng/mL. Patients without angioinvasion exhibited higher vaspin concentration (2.5 ng/mL), along with a range of values of 0.3–4.4 ng/mL. Median concentration of vaspin (1.2 ng/mL), along with a range of values from 0.1 to 3.1 ng/mL in patients with lymph node metastases was lower compared to the median serum vaspin concentration in patients without lymph node metastases (2.4 ng/mL) along with a range of values from 0.6 to 4.4 ng/mL. The level of statistical significance was *p* = 0.026. Results are presented in Table 6.

Median concentration of vaspin was significantly higher in patients of the study group with endometrial cancer compared to the median serum concentrations of vaspin in the non-cancer control group (*p* = 0.001). The median serum concentration in the control group was 3.1 ng/mL, along with a range of values from 0.7–6.6 ng/mL, while in the study group was 1.3 ng/mL, along with a range of values from 1.3 ng/mL to 2.9 ng/mL. Results are presented in Table 7.

### 3.4. Evaluation of Vaspin as a Risk Factor for Endometrial Cancer (EC)

Analyzing vaspin as an independent risk factor for endometrial cancer according to a multivariate logistic regression model; it was found that independent risk factors for EC are high BMI, WC > 100 cm and concomitant type 2 diabetes. High levels of vaspin do not count as risk factors. It can be considered here that OR 0.64 (odds ratio) and OR 0.69 provide indirect evidence for vaspin as a protective factor in the incidence of endometrial cancer. Results are presented in Table 8.

### 3.5. Evaluation of Patient Survival Using Kaplan-Meier Curves

**Figure 2:**
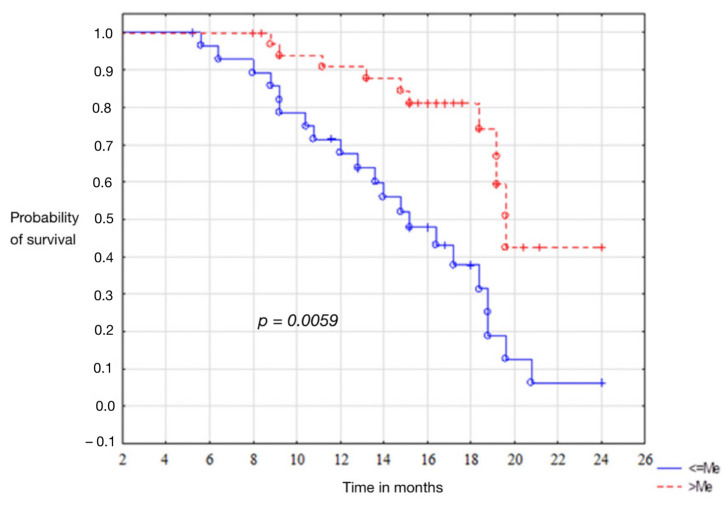
Kaplan-Meier curves for recurrence of endometrial cancer patients.

The average output levels of vaspin correlated with disease recurrence. Patients presenting values above the median were characterised by disease-free survival longer than 12.1 months. The level of statistical significance *p* = 0.0059. In the group of patients with endometrium cancer, the curves of Kaplan-Meier showed that the high baseline serum vaspin concentrations, were associated with the patient’s total survival time of 14.4 months. Unfortunately, the statistical significance level, *p* = 0.0521, has not been demonstrated. Results are presented in Figure 2.

### 3.6. Evaluation of Patient Survival Using Univariate and Multivariate Cox Regression

In our univariate and multivariate Cox analysis, we presented the impact of commonly recognised standard prognostic factors, as well as levels of serum vaspin concentrations affecting disease-free survival (DFS) and the time of total survival (OS). We found that among all selected factors, FIGO had the highest impact on DFS (HR-1.15 and HR-1.16), as well as vaspin concentrations related to the cut-off point (HR-0.77; HR-0.71). It was shown for these factors a level of significance. Median concentrations of vaspin and its concentration at the cut-off point had an impact on overall survival in both univariate and multivariate analysis for (*p* = 0.033, 0.041, 0.041, 0.049), respectively. Results are presented in Table 9.

### 3.7. Evaluation of Vaspin’s Tissue Expression by Immunohistochemical Method

Tissue expression of vaspin (serpin 12) was assessed in patients from the study group (endometrial cancer) and the control group (non-cancer control group) by immunohistochemistry (IHC). Estimates were made manually on IHC slides, according to the immunoreactivity score scale (IRS):
**0****Negative Result**1–6positive reaction7–12strong positive reaction

**Figure 3:**
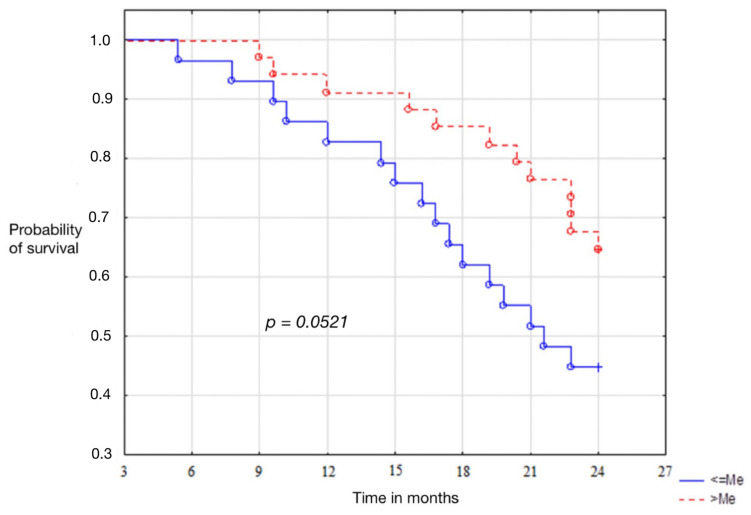
Kaplan-Meier curves for overall survival of patients with endometrial cancer.

**Figure 4:**
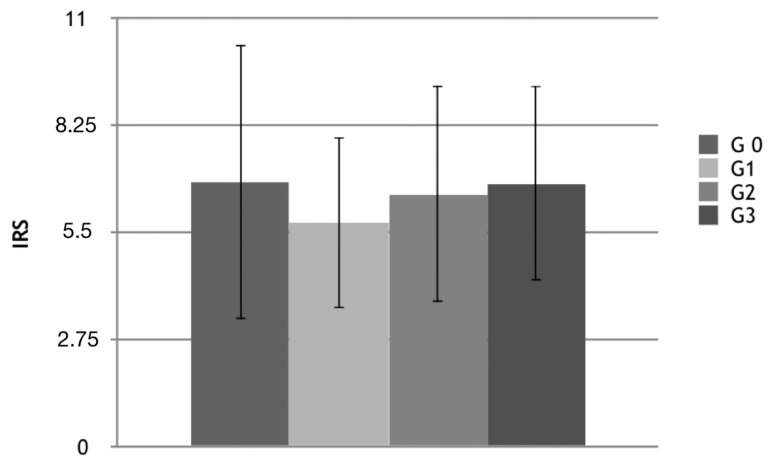
Evaluation by IRS scale of tissue vaspin expression in the study and control groups: IRS, Immunoreactive scale; G0–3, grading 0–3.

**Figure 5:**
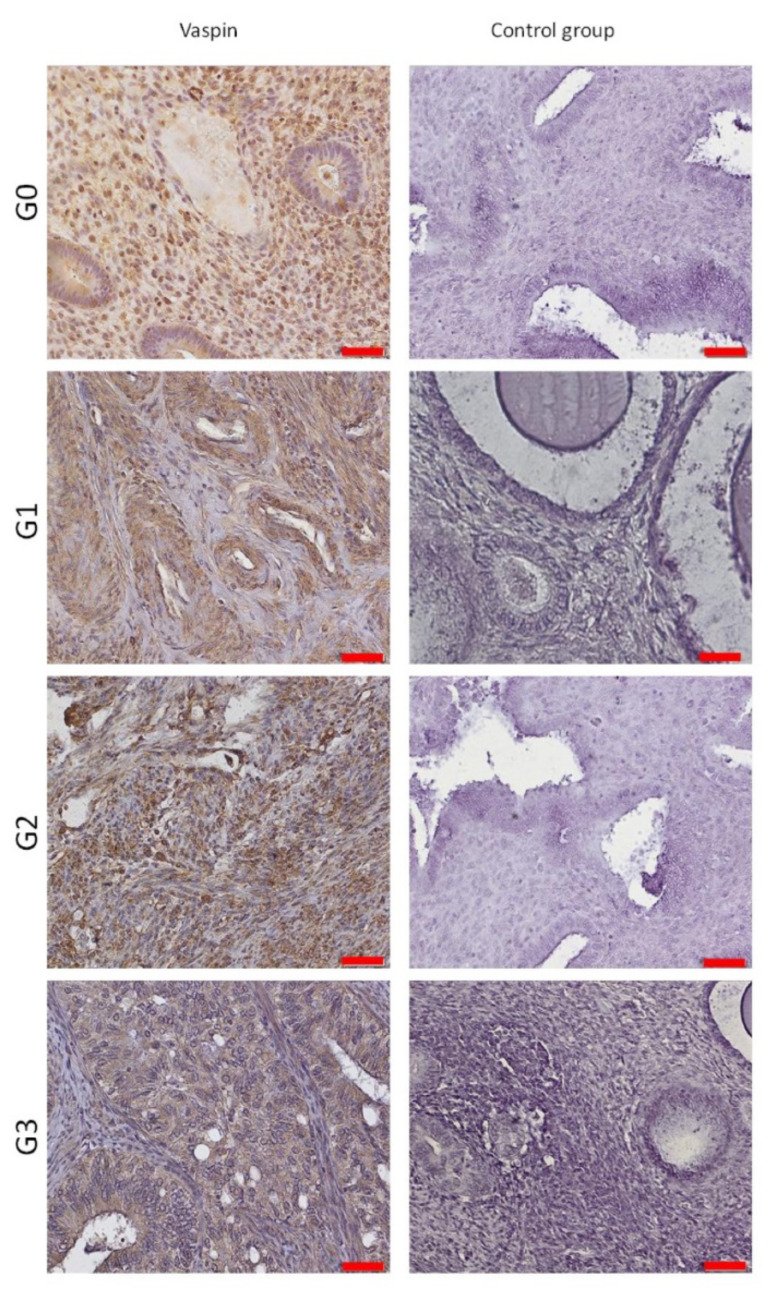
Tissue expression of vaspin in grading subgroups.

Positive and strong positive reaction to anti-serpin 12 antibody was located in G0 non-cancerous samples, but also in G1, G2, G3 test groups. Expression of vaspin varied between samples mainly in the control group from 0 to 7 (high SD, standard deviation). The positive reaction was mainly found in cytoplasm and cell membranes in pseudoustratified cylindrical epithelial cells (all scrapings containing epithelium) in both the study and control groups. The results are presented in Table 10 and Figure 3, Figure 4 and Figure 5.

## 4. Discussion

Obesity is now considered one of the most important diseases of civilization. It is a multidisciplinary sickness that increases the risk e.g., type 2 diabetes, insulin resistance, hyperlipidemia, hypertension, strokes and degenerative arthritis. It also predisposes to a number of cancers, in particular: colorectal cancer, breast cancer, and endometrial cancer [23,24]. In our study, the percentage of patients with obesity was highest with endometrial cancer, at 61.9%, compared to patients with endometrial polyps (23.8%) and endometrial myomas (14.3%). An analysis of risk factors showed that obesity is a factor in endometrial cancer.

A direct correlation between abnormal BMI values and the percentage risk of cancer was shown in a meta-analysis by the Agency for Research on Cancer (IARC). An increase of five BMI units results in a 50% increase in the risk of disease (RR-1.5; 95%CI-1.42–1.59). Mortality for obese women also increases, the relative risk for patients with BMI 30–34.9 is 2.53 [25] and with BMI > 40, the relative risk (RR) is 6.25. In endometrial cancer, increased production of hormones from adipose tissue, most notably androstenedione and testosterone, which aromatize to estrone and estradiol, cause continuous stimulation of the endometrium. Another risk factor for endometrial cancer included in our study was hypertension. The percentage of patients with hypertension in the group of endometrial cancers was 60.3%, while in the group of benign lesions: 17.6% with endometrial polyps and 22.1% with endometrial myomas. The pathomechanisms that would explain the adverse effects of hypertension on the risk of endometrial cancer are still unclear, despite the many studies that have been conducted. It seems that long-term hypertension may lead to cellular aging and inhibition of apoptosis [26,27,28]. It has also been suggested that drugs used to treat hypertension may increase the incidence of cancer. 

Analyzing carbohydrate metabolism in the study group, the percentage of female patients with type 2 diabetes is 51.4%. This is consistent with other reports which can be explained by the fact that excessive body weight and obesity are directly correlated with insulin resistance [12]. Cancer cells can respond to the activating effects of insulin through signal transduction pathways. Especially when serum insulin levels are high, as in obese patients with type 2 diabetes, IGF-1 (insulin-like growth factor) receptors are now thought to mediate main proliferative effects. In our study, 46.9% of patients with endometrial cancer had thyroid disease. Researchers have reported in numerous publications that hypothyroidism, in particular, increases the incidence of endometrial cancer. It should be noted that elevated TSH levels increase the incidence of metabolic syndrome. Seebacher et al., examined clinicopathological parameters in 199 patients with EC and found that TSH levels were not correlated with obesity, hypertension and carbohydrate disorders, but only with dyslipidemia [29,30]. Adipose tissue is a rich source of secreted polypeptides called adipokines, which are thought to regulate metabolism and participate in modifying chronic inflammation in patients with visceral obesity. There are a number of adipokines whose role is not yet understood. 

One of these is vaspin of the serpin family, produced by visceral adipose tissue with likely anti-inflammatory effects [31]. The effect on carbohydrate metabolism remains unclear. In our study, serum vaspin levels were statistically significantly different between optimal weight and overweight patients. Differences were also shown in serum concentrations between overweight and obese patients. Blüher et al., in their publication showed an association between vaspin with obesity and insulin resistance. They indicate that serum vaspin levels correlate with BMI. In our study, the correlation was also high at r = 0.658 [32]. Taking vaspin in obese mice improves glucose tolerance, insulin sensitivity and modifies the expression of genes responsible for insulin resistance. Klöting et al., showed that it led to a reduction in food intake and maintained its blood glucose-lowering effect [33]. It has also been discovered that increased oxidative stress after short- and long-term physical training reduces serum vaspin concentrations, while temporary changes in tissue insulin sensitivity do not appear to affect the regulation of serum vaspin concentrations. That is, it can be interpreted that physical activity has a very strong effect, through the regulation of oxidative stress [34]. To summarize the cardiovascular effects of vaspin, there have been a small number of reports showing similar results to our study, with no differences in serum vaspin levels in patients with heart disease or hypertension compared to women without these conditions. Most publications emphasize a beneficial role in inhibiting progressive atherosclerosis e.g., in the pathomechanism of various metabolic diseases, including diabetes [35,36,37]. 

The main purpose of our study was to investigate the relationship between serum concentrations and tissue expression of vaspin in patients with endometrial cancer. In our study, we found significantly lower levels of vaspin in patients with endometrial cancer compared to patients with benign endometrial lesions. Similar results were also observed when dividing the control group into subgroups according to histopathological diagnosis. In 2013, Erdogan et al., published a study conducted on 130 patients, 60 with endometrial cancer and 70 patients with benign endometrium [38]. They showed that median vaspin concentrations were lower in patients with endometrial cancer compared to controls (0.21 versus 0.39 ng/mL). Subgroup analysis of different percentiles of 50% CI, 75% CI and 95% CI (confidence interval) also confirmed lower vaspin concentrations compared to the control groups. In addition, we showed, using a multivariate regression model, that vaspin may be an independent factor protecting patients from developing endometrial cancer. In a study conducted by Cymbaluk-Ploska et al., published in 2018, statistically significantly lower serum vaspin levels were found in patients with endometrial cancer. The study was conducted on 168 patients, including 92 with confirmed endometrial cancer [22]. In our study, we found that in more advanced lesions, vaspin concentrations were lower compared to the median concentrations of patients with less advanced cancer. There were no differences according to the histopathological differentiation of the tumor. Only one publication presented data on serum vaspin concentrations according to FIGO. The researchers reported that patients with lymph node or lymphatic vessel involvement had lower serum vaspin levels, as did those with deeper infiltration of the myometrium [38]. Median levels of vaspin were also studied between different tumor grading levels and higher levels were noted [1.1 ng/mL] in patients with FIGO stage I and II endometrial cancer compared to patients with FIGO clinical stage III and IV (0.52 ng/mL) [22]. In the Erdogan et al., publication, patients were not divided into subgroups according to staging and grading, so we do not have comparative vaspin concentrations were not present [38]. 

In our study, we analyzed the association of preoperative serum concentrations in relation to recurrence and overall survival of patients. Median baseline vaspin levels correlated significantly with disease recurrence. Patients presenting values above the median were characterized by a disease-free time of 12.1 months longer. The results were statistically significant. The results were different in the group of endometrial cancer patients as to the association of serum vaspin concentrations with overall survival. Kaplan-Meier curves showed that high baseline concentrations were associated with a patient’s overall survival time longer by 14.4 months. Unfortunately, in this case, the level of statistical significance was not reached, *p* = 0.0521. No other publications are available that consider the possibility of using vaspin as a prognostic marker in endometrial cancer. 

Our own research indirectly confirms the role of vaspin as a modulator of insulin resistance, which often accompanies obese patients. The role of this protein is very important in the process of improving insulin sensitivity of tissues. It appears to be a protein that could be used in gynecology as an indicator protein in patients with abnormal endometrial bleeding. Further studies of vaspin as a biomarker to isolate patients at high risk of rapid recurrence remain to be considered. This is a group of women who require special attention because of the difficulty of treating recurrent endometrial cancer.

## 5. Conclusions

Vaspin serum concentrations may be independent prognostic and risk factors for endometrial cancer. Tissue expression of vaspin cannot be a histological marker to distinguish between cancer and non-cancerous lesions.

## Figures and Tables

**Table 1:** Division of endometrial cancer patients: G1–3, grading 1–3; FIGO, International Federation of Gynecology and Obstetrics.

Groups	Distribution	Patients, *n* (%)
**Histopathological grade of the tumor**	G1	18 (28%)
	G2	32 (49%)
	G3	15 (23%)
**Clinical stage of the tumor**	FIGO I and II	46 (71%)
	FIGO III and IV	19 (29%)
**Myometrial infiltration depth**	Superficial myometrial infiltration (<^1^/_2_ of the thickness)	37 (57%)
	Deep myometrial infiltration (>^1^/_2_ of the thickness)	28 (43%)
**Lymph node metastases**	Yes	19 (29%)
	No	46 (71%)
**LVSI (Lymphovascular space invasion) metastases**	Yes	39 (60%)
	No	26 (40%)
**Presence of angioinvasion**	Yes	30 (46%)
	No	35 (54%)

**Table 2:** Characteristics of patients divided into subgroups: BMI, body mass index; WC, waist circumference; HA, arterial hypertension; DM type 2, diabetes mellitus type 2.

Characteristics		Patients, *n* (%)
**Marital status**	widow	34 (27%)
	single	21 (17%)
	married	29 (23%)
	divorced	43 (34%)
**Education**	vocational education	32 (25%)
	secondary education	58 (46%)
	university education	37 (29%)
**Place of residence**	countryside	41 (32%)
	Cities with less than 100,000 inhabitants	57 (45%)
	City with more than 100,000 inhabitants	29 (23%)
**Hormonal state**	premenopausal state (PM)	40 (31%)
	postmenopausal state (M)	87 (69%)
**HA [>140/90 mmHg]**	yes	69 (54%)
	no	58 (46%)
**Fasting blood glucose [mg%]**	>110 mg%	93 (73%)
	<110 mg%	34 (27%)
**DM type 2**	yes	78 (57%)
	no	59 (43%)
**Thyroid diseases**	yes	81 (64%)
	no	46 (36%)
**BMI [kg/m^2^]**	<25	36 (28%)
	25–30	49 (39%)
	>30	42 (33%)
**WC [cm]**	>100	60 (47%)
	<100	67 (53%)

**Table 3:** Characteristics of patients: BMI, body mass index; WC, waist circumference; HA, arterial hypertension; DM type 2, diabetes mellitus type 2.

Diagnosis	Single		Married		*p*	Divorced		Widow		*p*
	*n*	%	*n*	%		*n*	%	*n*	%	
**Endometrial** **cancer**	9	42.9	18	62.1	0.006	21	48.8	17	50.0	NS
**Endometrial** **polyps**	10	47.6	5	17.2	0.045	7	16.3	6	17.6	NS
**Uterine myomas**	2	9.5	6	20.7	0.032	15	34.9	11	32.4	NS
	**Vocational education**		**Secondary education**		***p***	**University education**		**Secondary education**		***p***
	***n***	**%**	***n***	**%**		***n***	**%**	***n***	**%**	
**Endometrial** **cancer**	15	46.9	30	51.7	NS	20	54.1	30	51.7	NS
**Endometrial** **polyps**	12	37.5	12	20.7	0.036	6	16.2	12	20.7	NS
**Uterine myomas**	5	15.6	16	27.6	0.023	11	29.7	16	27.6	NS
	**Countryside**		**Cities with less than** **100,000 inhabitants**		***p***	**City with more than** **100,000 inhabitants**		**Cities with less than** **100,000 inhabitants**		***p***
	***n***	**%**	***n***	**%**		***n***	**%**	***n***	**%**	
**Endometrial cancer**	21	51.2	25	43.9	NS	19	65.5	25	43.9	NS
**Endometrial polyps**	9	22.0	15	26.3	0.031	6	20.7	15	26.3	0.035
**Uterine myomas**	11	26.8	17	29.8	0.042	4	13.8	17	29.8	0.038
	**Premenopausal state (PM)**				**Postmenopausal state (M)**				***p***	
	***n***		**%**		***n***		**%**			
**Endometrial cancer**	13		32.5		52		59.8		0.031	
**Endometrial polyps**	13		32.5		17		19.5		0.048	
**Uterine myomas**	14		35.0		18		79.3		NS	
	**HA—yes**				**HA—no**				***p***	
	***n***		**%**		***n***		**%**			
**Endometrial cancer**	41		60.3		24		40.7		0.033	
**Endometrial polyps**	12		17.6		18		30.5		0.046	
**Uterine myomas**	15		22.1		17		28.8		NS	
	**DM type 2—yes**				**DM type 2—no**				***p***	
	***n***		**%**		***n***		**%**			
**Endometrial cancer**	40		51.4		25		51.0		NS	
**Endometrial polyps**	19		24.3		11		22.5		NS	
**Uterine myomas**	19		24.3		13		26.5		NS	
	**Thyroid diseases—yes**				**Thyrois diseases—no**				***p***	
	***n***		**%**		***n***		**%**			
**Endometrial cancer**	38		46.9		27		64.2		0.042	
**Endometrial polyps**	22		27.2		8		19.0		0.009	
**Uterine myomas**	21		25.9		9		16.8		0.012	
	**WC < 100 cm**				**WC > 100 cm**				***p***	
	***n***		**%**		***n***		**%**			
**Endometrial cancer**	23		34.3		42		70.0		0.006	
**Endometrial polyps**	24		35.8		8		13.3		0.103	
**Uterine myomas**	20		29.9		10		16.7		0.018	
	**BMI < 25**		**BMI 25–30**		***p***	**BMI > 30**		**BMI 25–30**		***p***
	***n***	**%**	***n***	**%**		***n***	**%**	***n***	**%**	
**Endometrial cancer**	14	38.9	25	51.0	0.031	26	61.9	25	51.0	NS
**Endometrial polyps**	10	27.8	10	20.4	NS	10	23.8	10	20.4	NS
**Uterine myomas**	12	33.3	14	29.6	NS	6	14.3	14	29.6	0.024

**Table 4:** Based on the evaluation of serum vaspin levels in relation to subgroups division: BMI, body mass index.

Statistical Parameters	Vaspin [ng/mL]		*p*
	BMI < 25	BMI 25–30	
**Range**	0.3–3.3	0.8–5.6	0.038
**Median**	2.8	3.5	
**Confidence interval**	1.8–3.7	1.9–3.2	
**Standard deviation**	0.2	0.4	
	**Vaspin [ng/mL]**		***p***
	**BMI 25–30**	**BMI > 30**	
**Range**	0.8–5.6	3.3–6.6	0.012
**Median**	3.5	5.2	
**Confidence interval**	1.9–3.2	3.6–5.9	
**Standard deviation**	0.4	0.6	
	**Vaspin [ng/mL]**		***p***
	**BMI < 25**	**BMI > 30**	
**Range**	0.3–3.3	3.3–6.6	0.004
**Median**	2.8	5.2	
**Confidence interval**	1.8–3.7	3.6–5.9	
**Standard deviation**	0.2	0.6	

**Table 5:** Serum vaspin concentrations in female patients depending on WC: WC, waist circumference; HA, arterial hypertension; DM type 2, diabetes mellitus type 2.

Statistical Parameters	Vaspin [ng/mL]		*p*
	WC < 100 [cm]	WC > 100 [cm]	
**Range**	0.7–4.9	0.3–6.1	0.022
**Median**	3.5	5.5	
**Confidence interval**	2.4–4.1	2.8–4.2	
**Standard deviation**	0.3	0.4	
	**Vaspin [ng/mL]**		***p***
	**HA—yes**	**HA—no**	
**Range**	0.3–6.6	0.5–6.4	NS
**Median**	3.7	4.1	
**Confidence interval**	3.1–3.8	3.7–4.2	
**Standard deviation**	0.5	0.3	
	**Vaspin [ng/mL]**		***p***
	**DM type 2—yes**	**DM type 2—no**	
**Range**	0.3–5.9	0.4–6.6	NS
**Median**	5.0	5.1	
**Confidence interval**	2.1–5.1	2.7–4.8	
**Standard deviation**	0.4	0.3	

**Table 6:** Evaluation of serum vaspin levels in patients with endometrial cancer in relation to subgroups: G1–3, grading 1–3; FIGO, International Federation of Gynecology and Obstetrics; LVSI metastases, lymphovascular space invasion metastases.

Statistical Parameters	Vaspin [ng/mL]		*p*
	FIGO I, II	FIGO III, IV	
**Range**	0.4–3.8	0.1–2.6	0.041
**Median**	1.5	1.0	
**Confidence interval**	1.3–2.2	0.5–1.2	
**Standard deviation**	0.2	0.1	
	**Vaspin [ng/mL]**		***p***
	**G1**	**G2**	
**Range**	1.0–3.8	0.8–3.3	NS
**Median**	2.0	1.6	
**Confidence interval**	1.9–2.8	1.3–2.2	
**Standard deviation**	0.3	0.2	
	**Vaspin [ng/mL]**		***p***
	**G2**	**G3**	
**Range**	0.8–3.3	0.1–2.5	NS
**Median**	1.6	1.1	
**Confidence interval**	1.3–2.2	0.6–1.4	
**Standard deviation**	0.2	0.1	
	**Vaspin [ng/mL]**		***p***
	**G1**	**G3**	
**Range**	1.0–3.8	0.1–2.5	0.049
**Median**	2.0	1.1	
**Confidence interval**	1.9–2.8	0.6–1.4	
**Standard deviation**	2.2	0.1	
	**Vaspin [ng/mL]**		***p***
	**Superficial myometrial infiltration**	**Deep myometrial infiltration**	
**Range**	0.4–4.1	0.3–3.9	NS
**Median**	2.2	2.0	
**Confidence interval**	1.9–2.6	1.3–2.6	
**Standard deviation**	0.4	0.2	
	**Vaspin [ng/mL]**		***p***
	**LVSI metastases—yes**	**LVSI metastases—no**	
**Range**	0.3–4.1	0.5–4.4	0.037
**Median**	1.8	2.8	
**Confidence interval**	1.6–3.0	1.9–2.9	
**Standard deviation**	0.3	0.5	
	**Vaspin [ng/mL]**		***p***
	**Presence of angioinvasion—yes**	**Presence of angioinvasion—no**	
**Range**	0.1–3.5	0.3–4.4	NS
**Median**	1.5	2.5	
**Confidence interval**	1.6–2.2	2.1–2.5	
**Standard deviation**	0.2	0.4	
	**Vaspin [ng/mL]**		***p***
	**Lymph node metastases—yes**	**Lymph node metastases—no**	
**Range**	0.1–3.1	0.6–4.4	0.026
**Median**	1.2	2.4	
**Confidence interval**	0.8–1.4	1.8–2.7	
**Standard deviation**	0.1	0.4	

**Table 7:** Serum vaspin concentrations in patients from the study and control groups.

Statistical Parameters	Vaspin [ng/mL]		*p*
	Endometrial Cancer	Control Group	
**Range**	1.3–2.9	0.7–6.6	0.001
**Median**	1.3	3.1	
**Confidence interval**	0.8–1.9	2.8–3.8	
**Standard deviation**	0.1	0.3	

**Table 8:** Evaluation of vaspin as an independent risk factor for endometrial cancer according to the model of multivariate regression: BMI, body mass index; WC, waist circumference; WHR, waist-hip ratio; HA, arterial hypertension; DM type 2, diabetes mellitus type 2; OR, odds ratio; Cl, confidence interval; *p*, probability value.

	OR	95% Cl	*p*
**BMI**	1.3	1.18–1.39	0.032
**WC**	1.09	0.96–1.11	0.049
**Fasting blood glucose [mg%]**	0.93	0.9–1.10	0.163
**DM type 2**	1.17	1.04–1.21	0.022
**HA**	0.97	0.95–1.14	0.638
**WHR**	1.04	0.93–1.09	0.734
**Vaspin above median [ng/mL]**	0.69	0.65–0.71	0.047
**Vaspin above the cut-off point of 1.08 [ng/mL]**	0.64	0.59–0.65	0.014

**Table 9:** Evaluation of patient survival using univariate and multivariate Cox regression: DFS, disease-free survival; OS (overall survival); Cl, confidence interval; *p*, probability value.

Univariate Analysis						
Variables		DFS			OS	
	HR	95% Cl	*p*-Value	HR	95% Cl	*p*
**Age (above and below median)**	1.11	0.91–1.13	0.103	1.08	0.97–1.19	0.061
**FIGO (III and IV vs I and II)**	1.15	1.05–1.18	0.041	1.13	1.05–1.16	0.053
**Grade (G3 vs G1)**	1.14	1.09–1.20	0.079	1.11	1.10–1.19	0.066
**Vaspin concentration (above and below median)**	0.78	0.70–0.84	0.044	0.82	0.79–0.84	0.033
**Vaspin concentration (above and below** **95% Cl)**	0.85	0.81–0.86	0.138	0.79	0.75–0.91	0.088
**Vaspin concentration (above and below the cut-off point)**	0.77	0.70–0.78	0.048	0.84	0.80–0.92	0.041
**Multivariate Analysis**						
		**DFS**			**OS**	
	**HR**	**95% Cl**	***p*** **-Value**	**HR**	**95% Cl**	***p***
**Age (above and below median)**	1.08	1.05–1.13	0.085	1.12	1.08–1.16	0.056
**FIGO (III and IV vs I and II)**	1.16	1.13–1.19	0.048	1.14	1.10–1.18	0.031
**Grade (G3 vs G1)**	1.04	1.02–1.10	0.097	1.07	0.98–1.15	0.086
**Vaspin concentration (above and below median)**	0.86	0.68–0.75	0.021	0.84	0.80–0.90	0.041
**Vaspin concentration (above and below** **95% Cl)**	0.78	0.73–0.80	0.326	0.81	0.77–0.84	0.148
**Vaspin concentration (above and below the cut-off point)**	0.71	0.64–0.75	0.025	0.90	0.89–0.96	0.049

**Table 10:** Evaluation of vaspin expression in the control group and the study group according to the histopathological differentiation of the tumor: IRS, Immunoreactive scale; SD, standard deviation.

Groups	IRS Average	SD
**G0**	6.82	±3.512364
**G1**	5.77	±2.177247
**G2**	6.51	±2.771245
**G3**	6.57	± 2.495795

## Data Availability

The data presented in this study are available on request from the corresponding author, D.P., upon reasonable request.
